# Draft Genome Sequence of *Methanoculleus sediminis* S3Fa^T^, a Hydrogenotrophic Methanogen Isolated from a Submarine Mud Volcano in Taiwan

**DOI:** 10.1128/genomeA.00308-16

**Published:** 2016-04-21

**Authors:** Sheng-Chung Chen, Mei-Fei Chen, Chieh-Yin Weng, Mei-Chin Lai, Sue-Yao Wu

**Affiliations:** a Department of Life Sciences, National Chung Hsing University, Taichung, Taiwan; b Agricultural Biotechnology Center, National Chung Hsing University, Taichung, Taiwan

## Abstract

Here, we announce the genome sequence of *Methanoculleus sediminis* S3Fa^T^ (DSM 29354^T^), a strict anaerobic methanoarchaeon, which was isolated from sediments near the submarine mud volcano MV4 located offshore in southwestern Taiwan. The 2.49-Mb genome consists of 2,459 predicted genes, 3 rRNAs, 48 tRNAs, and 1 ncRNA. The sequence of this novel strain may provide more information for species delineation and the roles that this strain plays in the unique marine mud volcano habitat.

## GENOME ANNOUNCEMENT

*Methanoculleus sediminis* S3Fa^T^, a mesophilic and hydrogenotrophic methanogen, was isolated from sediments near the submarine mud volcano MV4 located offshore in southwestern Taiwan and described as a novel species within the genus *Methanoculleus* (1). The genus *Methanoculleus* currently comprises 11 characterized and valid species: *M. marisnigri* JR1^T^ (2), *M. submarinus* Nankai-1^T^ (3), *M. chikugoensis* MG62^T^ (4), *M. thermophiles* CR-1^T^ (5), *M. palmolei* INSLUZ^T^ (6), *M. bourgensis* MS2^T^ (7), *M. receptaculi* ZC-2^T^ (8), *M. hydrogenitrophicus* HC^T^ (9), *M. horonobensis* T10^T^ (10), *M. taiwanensis* CYW4^T^ (11), and *M. sediminis* S3Fa^T^ (1). The sources of these *Methanoculleus* spp. are truly diverse, including marine sediments, wastewater plants, wetlands, oil fields, and paddy fields. Here, we provide the draft genome sequence of *M. sediminis* S3Fa^T^ as a basis for future comparative studies and understanding the ecological roles of this genus.

Whole-genome shotgun sequencing was performed using the Illumina MiSeq platform with 2 × 300-bp reads. The reads were quality-filtered, trimmed, and assembled into contigs using the *de novo* assembler CLC Genomic Workbench version 7.5. The draft genome comprises 15 contigs with total length of ~2.49-Mb and a G+C content of 62.3%. Gene annotations were performed by using the NCBI Prokaryotic Genome Automatic Annotation Pipeline (PGAAP); 2,459 predicted genes, 3 rRNAs, 48 tRNAs, and 1 ncRNA were obtained.

The genome contains the genes of formate dehydrogenase, which is essential for formate utilization for methane production and growth of *Methanoculleus sediminis* S3Fa^T^. Three clusters of regularly interspaced short palindromic repeat (CRISPR)-associated *cas* genes were identified. Furthermore, this genome contains three coding genes for trehalose synthases, which convert ADP-glucose to trehalose. This genome also comprises complete gene sets of heat shock proteins, including DnaK, DnaJ, GrpE, GroEL, GroES, prefoldin subunits, and small heat shock proteins. The genetic and physiological characteristics of strain S3Fa^T^ will be unveiled by comparative genomic analyses with *Methanoculleus* spp. and methanogens within other taxa.

### Nucleotide sequence accession numbers.

This whole-genome shotgun project has been deposited at DDBJ/EMBL/GenBank under the accession number JXOJ00000000. The version described in this paper is the first version, JXOJ01000000.

## References

[1] Chen SC, Chen MF, Lai MC, Weng CY, Wu SY, Lin S, Yang TF, Chen PC 2015 *Methanoculleus sediminis* sp. nov., a methanogen from sediments near a submarine mud volcano. Int J Syst Evol Microbiol 65:2141–2147. doi:10.1099/ijs.0.000233. 25855623

[2] Romesser JA, Wolfe RS, Mayer F, Spiess E, Walther-Mauruschat A 1979 *Methanogenium*, a new genus of marine methanogenic bacteria, and characterization of *Methanogenium cariaci* sp. nov. and *Methanogenium marisnigri* sp. nov. Arch Microbiol 121:147–153. doi:10.1007/BF00689979.

[3] Mikucki JA, Liu Y, Delwiche M, Colwell FS, Boone DR 2003 Isolation of a methanogen from deep marine sediments that contain methane hydrates, and description of *Methanoculleus submarinus* sp. nov. Appl Environ Microbiol 69:3311–3316. doi:10.1128/AEM.69.6.3311-3316.2003. 12788731 PMC161549

[4] Dianou D, Miyaki T, Asakawa S, Morii H, Nagaoka K, Oyaizu H, Matsumoto S 2001 *Methanoculleus chikugoensis* sp. nov., a novel methanogenic archaeon isolated from paddy field soil in Japan, and DNA-DNA hybridization among *Methanoculleus* species. Int J Syst Evol Microbiol 51:1663–1669. doi:10.1099/00207713-51-5-1663. 11594593

[5] Rivard CJ, Smith PH 1982 Isolation and characterization of a thermophilic marine methanogenic bacterium, *Methanogenium thermophilicum* sp. nov. Int J Syst Bacteriol 32:430–436. doi:10.1099/00207713-32-4-430.

[6] Zellner G, Messner P, Winter J, Stackebrandt E 1998 *Methanoculleus palmolei* sp. nov., an irregularly coccoid methanogen from an anaerobic digester treating wastewater of a palm oil plant in North-Sumatra, Indonesia. Int J Syst Bacteriol 48:1111–1117. doi:10.1099/00207713-48-4-1111. 9828413

[7] Ollivier BM, Mah RA, Garcia JL, Boone DR 1986 Isolation and characterization of *Methanogenium bourgense* sp. nov. Int J Syst Bacteriol 36:297–301. doi:10.1099/00207713-36-2-297.

[8] Cheng L, Qiu TL, Li X, Wang WD, Deng Y, Yin XB, Zhang H 2008 Isolation and characterization of *Methanoculleus receptaculi* sp. nov. from Shengli oil field, China. FEMS Microbiol Lett 285:65–71. doi:10.1111/j.1574-6968.2008.01212.x. 18557787

[9] Tian J, Wang Y, Dong X 2010 *Methanoculleus hydrogenitrophicus* sp. nov., a methanogenic archaeon isolated from wetland soil. Int J Syst Evol Microbiol 60:2165–2169. doi:10.1099/ijs.0.019273-0. 19897615

[10] Shimizu S, Ueno A, Tamamura S, Naganuma T, Kaneko K 2013 *Methanoculleus horonobensis* sp. nov., a methanogenic archaeon isolated from a deep diatomaceous shale formation. Int J Syst Evol Microbiol 63:4320–4323. doi:10.1099/ijs.0.053520-0. 23832970

[11] Weng CY, Chen SC, Lai MC, Wu SY, Lin S, Yang TF, Chen PC 2015 *Methanoculleus taiwanensis* sp. nov., a methanogen isolated from deep marine sediment at the deformation front area near Taiwan. Int J Syst Evol Microbiol 65:1044–1049. doi:10.1099/ijs.0.000062. 25575827

